# *Cassia fistula* nutrition rich flower tea derived biotic nanoparticles synthesis, characterization and their antioxidant and anti-hyperglycaemic properties

**DOI:** 10.4314/ahs.v22i1.47

**Published:** 2022-03

**Authors:** Chinnadurai Veeramani, Ahmed S El Newehy, Mohammed A Alsaif, Khalid S Al-Numair

**Affiliations:** Department of Community Health Sciences, College of Applied Medical Sciences, King Saud University, P.O. Box 10219, Riyadh 11433, Saudi Arabia

**Keywords:** Nanoparticles, drug delivery, iron III chloride, Cassia fistula, glucose mechanism

## Abstract

**Background:**

*Cassia fistula* (CF) is a nutrient-rich flowering plant and it has been used to cure numerous human health problems including cardiac diseases, bacterial infection, and inflammation.

**Objective:**

The purpose of this study was to investigate the production and characterisation of biomimetic iron oxide nanoparticles (ICF) derived from CF flower tea as well as evaluate their antioxidant and anti-hyperglycemic properties.

**Methodology:**

CF tea derived ICF synthesis and characterized by established physical-chemical methods. Moreover, this synthesized ICF were checked for their antioxidant and anti-hyperglycemic properties such as alpha-amylase, glucose intake, total antioxidant (TAA), ferrous reducing (FA), and radical scavenging (DPPH) properties.

**Results:**

The synthesized ICF characterization and size were confirmed primarily by described physical and chemical methods. Our findings revealed that ICF have a powerful antihyperglycemic mechanism by involving alpha-amylase inhibition and enhanced glucose absorption. Meanwhile, this ICF exhibited distinguished antioxidant competence by improving TAA and free radical scavenging (TAA, DPPH) properties. Finally, this ICF has proven anti-hyperglycemic and antioxidant mechanisms due to their presence of nano-sized biomolecules.

**Conclusion:**

In this study, it might be concluded that the CF is the best source for iron oxide nanoparticles production with clarity, small size and high solidity. Moreover, this nanoparticle has proven in vitro anti-hyperglycemic and antioxidant mechanisms.

## Introduction

Diabetes mellitus is a clutch of metabolic disorders and a serious problem in current society because day by day, affected people have increased in developing and developed countries, even in Saudi Arabia. It is affected by numerous causes including metabolic imbalance of carbohydrates, protein, and fat, oxidative stress associated damage, autoimmune reaction, obesity, family history, and pancreatitis. Oxidative stress or free radicals are the most dangerous free elements for the development of diabetic complications, including cardiovascular and microvascular complications. Numerous biochemical pathways, including the polyol pathway, glucose autooxidation, and protein glycosylation, are mainly convoluted by the production of free radicals during prolonged uncontrolled hyperglycemia status. Numerous studies have proved that control of blood glucose is most effective for reducing diabetic complications, but even the optimal range of blood glucose control to prevent complications is still not yet proven by scientists, therefore, other drug approaches are needed. 1,2 Several studies have proved that hyperglycemia is mainly induced by increased generation of oxidative stress or free radicals. Therefore, it has been proven that antioxidant mediated drugs might be the most powerful and effective approach to preventing diabetic mediated complications. The plant sources or plant source derivatives associated with nanoparticles are considered as a novel therapy due to their natural care, healthier effectiveness without any side impact, simple way of synthesis and, most importantly less cost. 3,4

Nanotechnology is the most advanced technology and it has numerous applications, including several industrial fields and also in human health drug developments because of its nanosize, it has several positive approaches. Nanotechnology has been designated as the next revolution to hit many industries and it can be achieved accredited by scientists to the next step with the existing performance. It is a novel and innovative technology due to its major applications in the current fields of medicine, water purification, genetic engineering, and progressive microscopy methods. Presently, an enormous methods of nanoparticle synthesis are available with unique properties, including chemical, physical, and biological methods. 5,6 Recently, organisms associated with the synthesis of nanoparticles are fascinated by researchers for alternating chemical synthetic methods due to their practical worth, simplicity and non-toxic nature. 6,7 A variety of micro and macro organisms such as algae, fungi, bacteria, sea products, and plant sources are employed in the synthesis of nanoparticles because redox reactions occur in biological processes due to the reductive ability of cellular or extracellular constituents. The plant sources are the most plentiful and attracted by researchers due to their non-hazardous ingredientsf polyphenols, flavonoids, proteins, vitamins, carbohydrates, and other individual or groups of metabolites. 6,7,8 And also, mainly, these plant ingredients contribute electrons to the metals which are converted into zero charge metallic form at nanometric sizes. Recently, several chemicals have been used successfully for the synthesis of plant associated nanoparticles such as silver, gold, zinc oxide, and iron oxide. 7,9 Currently, among them, iron oxide nanoparticles have developed significantly due to their admirable magnification power and medicinal application. 10

Cassia fistula is a flowering plant and it is generally known as the golden flower. Cassia fistula has been recommended for curing many diseases, including skin and liver problems like liver disorder, fatty liver, jaundice, and bronchitis. Moreover, it has been used to control or cure many problems, such as neurodegenerative diseases, bacterial infection, and inflammation. 11,12 In Ayurvedic medicine, it is called “disease killer” and is used to solve many problems, such as ulcers, skin diseases, jaundice, burning sensations, malaria, rheumatism, syphilis, abdominal pain, constipation, and fever. Cassia fistula whole plant contain several chemical ingredients such as vitamin C and E, linoleic acid, glutamic acid, phenylalanine, proline, oxalic acids, tannins, fistulin, 4-Hydroxy benzoic acid hydrate, Phytol, 2-hexadecanone, and kaempferol etc... 13 Researchers have not yet examined the biological and chemical synthesis of iron oxide nanoparticles using the Cassia fistula and iron III chloride. Iron oxide is a naturally occurring mineral component that is non-toxic. It features more reactive surfaces and a greater high surface area. Iron oxides are used in a range of biomedical applications and have unique therapeutic qualities. As a result, we chose biotic approach synthesis for its potential clinical benefits. However, we have chosen this Cassia fistula flower to synthesize iron oxide nanoparticles. These chemical associated biological nanoparticles were characterized by stable physical and chemical methods and it was confirmed the nanoparticle size with high stability and limpidity. Hence, currently, researchers are focusing on identifying new novel drugs in natural ways to cure or control diabetes without any side effects or complications. Cassia fistula flower is used in Ayurvedic or Siddha medicine in many countries, including India and Saudi Arabia, to control diabetes, and this flower is recommended by Siddha doctors in a natural way as a green tea. As a result, we intend to look into the biological and chemical aspects of the synthesis of iron oxide nanoparticles using Cassia fistula flower tea and iron III chloride in this work. In addition, these synthesized nanoparticles were checked for their antioxidant and anti-hyperglycemic mechanisms.

## Methods

### Plant gathering and extraction

Cassia fistula fresh flowers were brought from Riyadh, Saudi Arabia (winder season of January month) and were authenticated botanically from Botany department at King Saud University. The calmed flowers were washed exhaustively with iron free water twice and removed impurities and dust. The washed flowers were dried naturally at room temperature for 3 days, then the flowers made powder. 500 mg of power was immersed in 100 mL of iron-free hot water and socked for 5 mins. The tea extract was filtered and this extract was used for nanoparticle fabrication.

### Nanoparticles fabrication method

The nanoparticles were fabricated by a modest co-precipitation method with a mixture of Cassia fistula flower extract and iron III chloride hexahydrate (FeCl3.6H2O). In the first step, an iron III chloride hexahydrate (Fe-Cl3.6H2O) solution was prepared by mixing 1.10 g of iron III chloride hexahydrate (FeCl3.6H2O) in 100 mL of iron free water underneath a nitrogen blanket. This iron III chloride solution was warmed at 80°C for 10 mins by using a magnetic stirrer. After 10 mins, 20 mL of extract was added slowly drop by drop and flowingly added sodium hydroxide until the block color was shown. The whole process occurs under the warm stirring of a magnetic stirrer. The block color shown was assured of the nanoparticle fabrication. This nanoparticle fabrication was cooled at a normal environment temperature for 30 mins and the formed supernatant was discorded. The pellet particles were eroded three times by deionized water with centrifugation at 10000 rpm for 15 mins and removed residual salts. Finally, the particles were dried overnight in an air-dry oven at 37 °C. This powder form of block colored nanoparticles was used for further characterization and to test biological activities.

### Characterization

Nanoparticles Surface Plasmon resonances were appraised by a UV-Visible spectrophotometer model and the range of 300 to 700 nm. The nanoparticle size was measured by transmission electron microscopy (TEM) analysis and the elements were measured by energy-dispersive X-ray spectroscopy (EDX) analysis. Nanoparticles zeta potential and distribution were measured by the dynamic light scattering (DLS) method. plant source ingredients overlapped with nanoparticles were identified by fourier transform infrared spectroscopy (FTIR) analysis. 14

### α-amylase restraint test

α-amylase assay of biologically synthesized nanoparticles was estimated by established methods with slight alterations. 15 0.25, 0.5, 1 ml of biologically synthesized nanoparticles were assorted with 0.5 ml of α-amylase solution. Afterwards, this mixture was kept at room temperature for 10 min. It was mixed with 0.5 mL of starch solution and left at room temperature for 10 minutes. Finally, 1 mL of dinitrosalicylic acid color reagent was added to stop the process. The mixtures were kept in water at 100 °C for 5 mins, then they were kept cooling until normal temperature. The mixture was diluted with 10 mL of iron-free water. The mixture absorbance was measured at 540 nm. The α-amylase inhibition was determined by using the below given formula and acarbose was used as a reference drug:

α-Amylase inhibition (%) = (Abscontrol−Abssample)/(Abscontrol)×100

Abs control represents the reaction mixture with added buffer instead of nanoparticles; Abssample represents the mixture with nanoparticles.

### Glucose intake in yeast cells

The glucose intake test was achieved in yeast cells by the method of Cirillo et al. 16 The Saccharomyces cerevisiae yeast (10% (v/v) with distilled water) suspension was prepared by centrifugation at 12000 ×g for 15 mins. Different amounts of biologically produced nanoparticles (25 to 200 g/ml) were combined with 1 mL of glucose (5 mM) solution and incubated for 10 minutes at room temperature. After that, 100 µl of the prepared yeast solution was added to the mixture, which was then allowed to sit at room temperature for 1 hour. After 1 hr, the mixtures were centrifuged at 12000 ×g for 15 mins and, lastly, the supernatant was used to estimate the glucose amount. Metronidazole was used as a reference drug. The glucose intake rate in yeast cells was measured by using the bellow formula.

Glucose intake calculation (%) = (Abscontrol—Abssample)/(Abscontrol)×100

### Antioxidant potential assay of nanoparticles

The total antioxidant action (TAA) of synthesized nanoparticles was examined by the Prieto et al. method. 17 The ferrous reducing activity (FA) of synthesized nanoparticles was assessed by the Oyaizu method. 18 Radical scavenging activity (DPPH) of the synthesized nanoparticles was assessed by Blois 19 and Desmarchelier et al. method. 20

### Statistical analysis

All tests were done three times (n= 3) and the data was given as a mean average of 3 readings ± average error. The significance between the mean average of separate groups was examined by SPSS software and the significance was noticed at (p < 0.05).

## Results

### Characterization of nanoparticles

**Figure 1:**
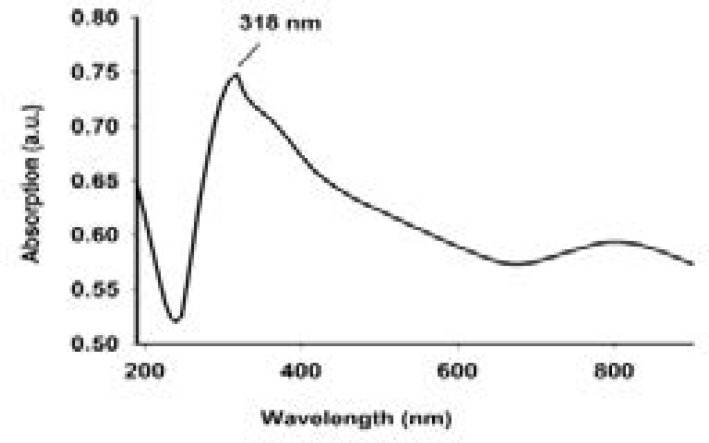
shown the absorption peak of CFNPs at different wavelength

**Figure 2:**
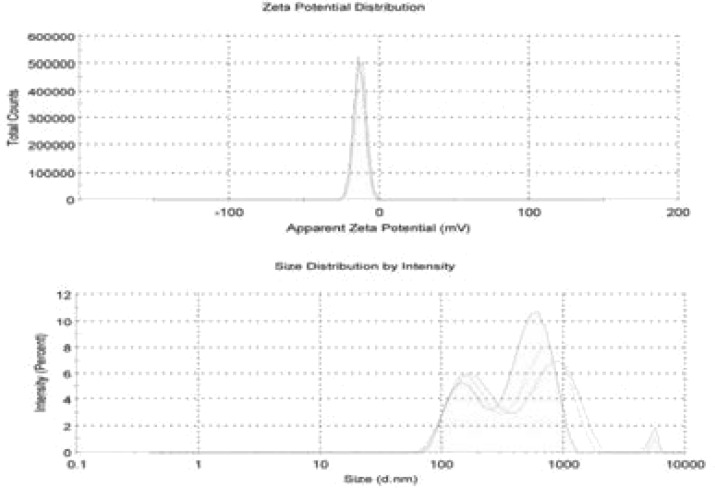
shown the seta potential and size distribution spectrum of CFNPs

**Figure 3:**
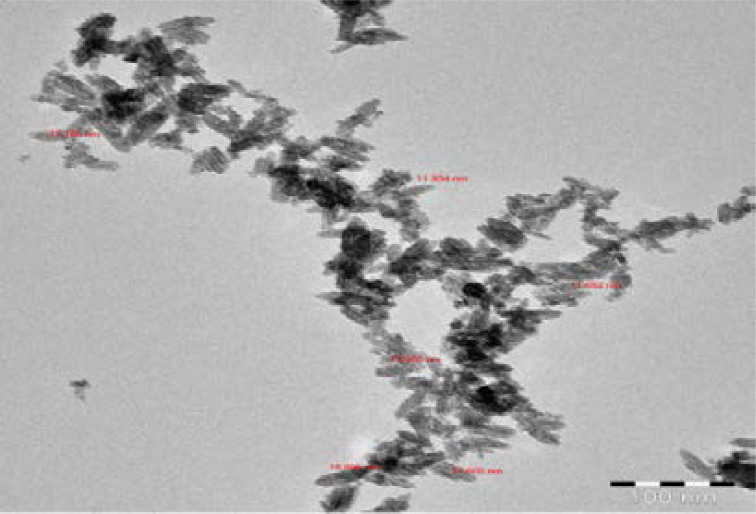
shown the TEM morphology of CFNPs

**Figure 4:**
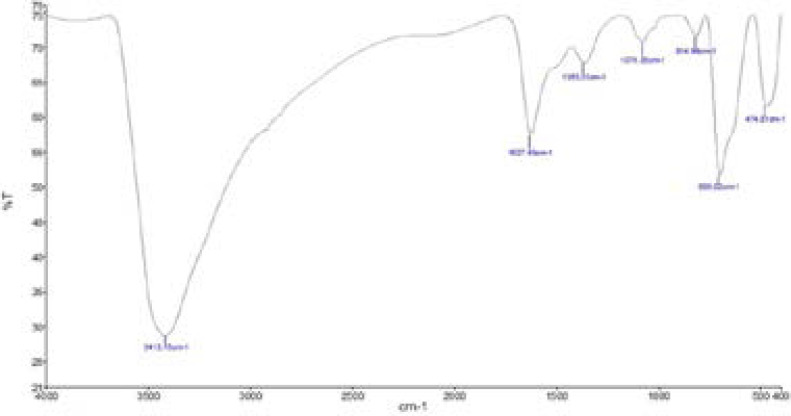
shown the FTIR peak of CFNPs

**Figure 5:**
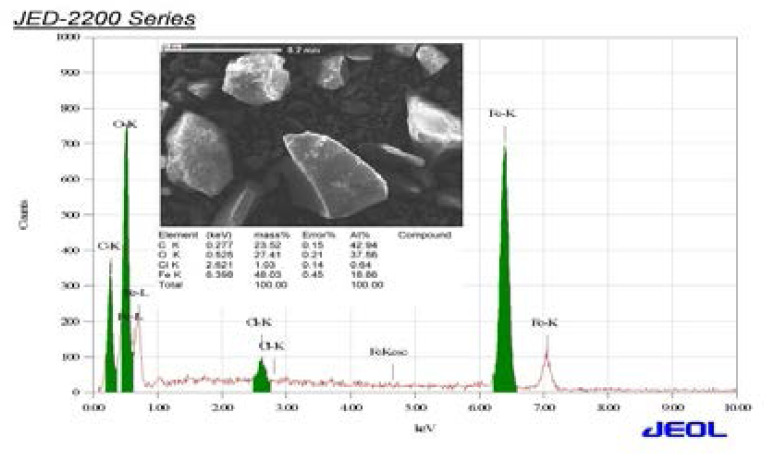
shown the elements determination of CFNPs by EDX spectrum

The synthesized iron nanoparticles from Cassia fistula flower tea were characterized by several described physical and chemical methods and these results are shown in Fig. 1 to 5. The synthesized nanoparticles were confirmed primarily by the absorption spectrum of exterior plasmon resonance at 318 nm. Additionally, it was confirmed by the size measurement of about 11.305 to 17.605 nm by the TEM method and the mean average size distribution value of 550.7 d.nm and the zeta potential value of-13.1 mV by the DLS method. The nanoparticles ingredients overlapped with the iron and this was confirmed by the FTIR method. Lastly, these biomolecules coincided with iron and this was confirmed by EDX spectrum Fe majorly present.

### Anti-hyperglycemic activity of nanoparticles

**Figure 6:**
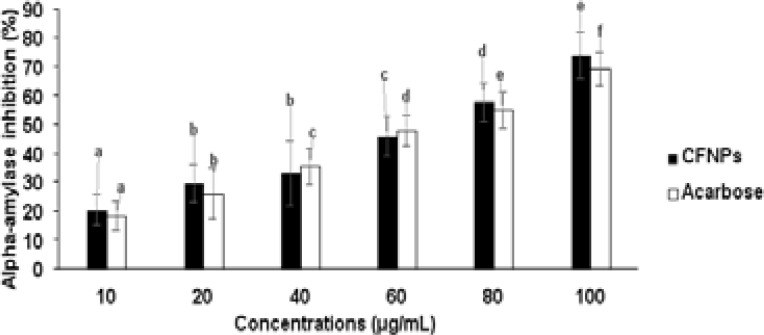
Shown the alpha-amylase reticence activity (%) of CFNPs All tests were done in tree times (n= 3) and the data were given as mean average of 3 reading ± average error. Significances between the means average of separate groups were examined by SPSS software and the significance noticed at the p < 0.05.

**Figure 7:**
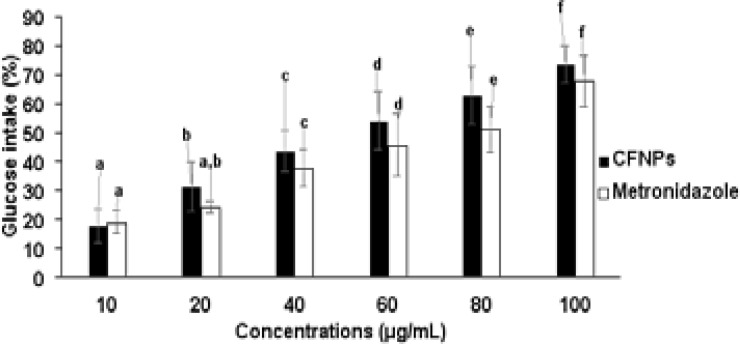
Shown the glucose intake calculation (%) of CFNPs All tests were done in tree times (n= 3) and the data were given as mean average of 3 reading ± average error. Significances between the means average of separate groups were examined by SPSS software and the significance noticed at the p < 0.05.

α-amylase inhibitory activity and glucose intake in yeast cells were tested with iron oxide nanoparticles and the results are shown in figures 6 and 7. The iron oxide nanoparticles showed greater inhibitory activity and a higher percentage of glucose intake in yeast cells near to standard drug, which could be a potential therapy for postprandial hyperglycemia.

### antioxidant properties of Nanoparticles

**Figure 8:**
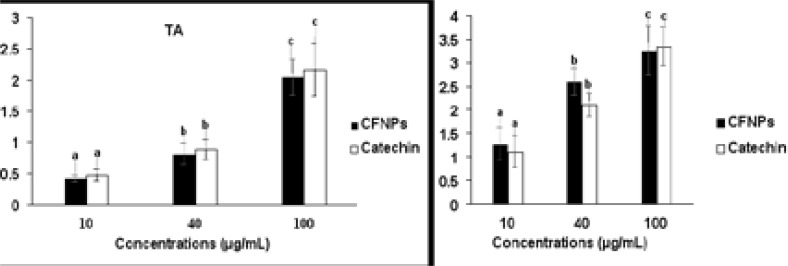
Shown the total antioxidant (TAA) and reducing activity (FA) of CFNPs All tests were done in tree times (n= 3) and the data were given as mean average of 3 reading ± standard deviation

**Figure 9:**
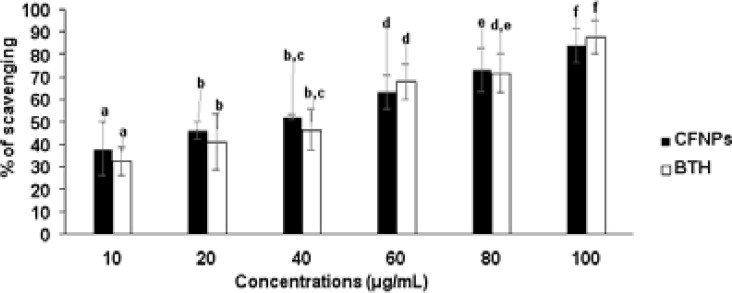
Shown the radical scavenging properties of CFNPs by DPHH method All tests were done in tree times (n= 3) and the data were given as mean average of 3 reading ± average error. Significances between the means average of separate groups were examined by SPSS software and the significance noticed at the p < 0.05.

The TAA, FA, and DPPH methods were used to test the antioxidant mechanisms of iron oxide nanoparticles, and the findings are presented in figs 8 and 9. The overall antioxidant potential of manufactured nanoparticles was found to grow dramatically in a dose-dependent manner, with findings that were similar to those of a standard drug. This obtained antioxidant activity of synthesized nanoparticles with minimal dosage concentrations may be associated with the nano-sized particles of plant extract ingredients. Depending on the dose concentrations, the FA reducing and DPPH radical scavenging activities of produced nanoparticles increased dramatically. The antioxidant mechanism has been proven by the scavenging activities of nanoparticles, which could be attributed to the presence of biomolecules in nanoparticles.

## Discussion

Iron oxide has been focused by scientists as a multiuse nanomaterial due to its non-toxic, ecofriendly, unique properties in physiological and chemical nature, and huge biological applications such as antidiabetic, anticancer, and antimicrobial activities. 21,22 Currently, biologically mixed synthesized iron oxide nanoparticles have achieved unique and valuable medicinal properties in several fields including diabetes, hypertension, cancer, and microbial infection. 21,22 Hence, in this study, we have planned to synthesize and characterize the biological associated iron oxide nanoparticles from Cassia fistula flower tea extract and iron III chloride. The iron nanoparticle ultraviolet region spectrum was observed at 318 nm, which is characteristic of surface plasmon resonance of metal free electrons into iron nanoparticles. Our ultraviolet region spectrum study primarily confirmed that the Fe III chloride may act as a metallic core and the used plant flower extract constituents such as polyphenols, flavonoids, vitamins, and terpinoids may act as a capping or reducing agent and the result is a zero charge of metallic nanoparticles. The synthesized iron nanoparticles from extract were used for measurement of zeta potential and size distribution by DLS methods. It is observed that the flower extract synthesized nanoparticles show a very narrow size distribution and an average size distribution value of 550.7 d.nm and a zeta potential value of-13.1 mV. However, this size distribution results in further evidence of the zero charge of metallic nanoparticles.

TEM is a modern ultrasensitive technology and it is used to pinpoint nanoparticle size and morphology. In our study, the synthesized nanoparticle size was assessed by TEM analysis. The nanoparticle size was shown to be spherical in shape with a diameter of 11.305 to 17.605 nm. This result is closely associated with the obtained size distribution by the DLS method, which could confirm the nanoparticles synthesized at a minimal size and purity. The interactions between the nanomaterial chemicals and the extracted constituents were measured by FTIR spectroscopy. The FTIR bands at 3413.15, 1627.40, 1363.03, 1076.35, 814.96, 699.00, and 474.81 cm-1 are represented by the O-H stretching, carbonyl group, C=N stretching, C-O-C phenolic hydroxyl group and Fe stretching. The metal free electrons into iron nanoparticles synthesis were confirmed by the FTIR method because the strong attraction of the presented biological compounds from extract overlapped with the free electrons from iron III chloride. Lastly, the synthesized nanoparticle elements were appraised and recognized by the EDX method. The biomolecules of Cassia fistula flower extract coincide with iron and this was confirmed by element analysis by EDX. In these results, Fe is majorly present and other Cl. C, and O are also present. As a result, it's concluded that Cassia fistula flower extract is a good source for iron nanoparticle synthesis with high purity, small size, and solidity.

Diabetes mellitus is a metabolic disorder and it is prevalently increasing in many countries due to lifestyle modifications. Even in this modern society, effective medicine without any side effects is very challenging and still being searched by scientists. Currently, the majority of research has started to find medicine from natural plant sources associated with nanotechnology due to their lower toxicity, lower cost and effectiveness. 8,23,24 Therefore, in our study, we have planned to investigate in vitro assays such as alpha-amylase and glucose intake for the anti-diabetic effectiveness of synthesized iron oxide nanoparticles from Cassia fistula flower extract and iron III chloride. Alpha-amylase is a digestive intestinal enzyme which is primarily associated with the digestion and absorption of carbohydrates. The Alpha-amylase inhibitor is important for carbohydrate deferral in the small intestine and for lowering postprandial blood glucose levels. Iron oxide nanoparticles were utilized in our investigation to examine α-amylase inhibitory activity, and they demonstrated higher inhibitory activity. Therefore, we are suggesting that this could be a possible therapeutic for postprandial hyperglycemia. Numerous studies have demonstrated that the majority of alpha-amylase inhibitors are isolated from plant sources. 25,26 The presence of single or complex nanosized biomolecules may reflect the inhibitory impact of biologically produced iron oxide nanoparticles. Sustained blood glucose control in diabetic patients plays an essential role in inhibiting complications and mortality. Glucose intake disorders in peripheral tissues are mainly associated with pathophysiological conditions such as obesity and insulin resistance. Glucose intake enhancer drugs in cells are important in diabetic patients, especially in type 2. In our study, nanoparticles with different concentrations were used to analyse in vitro glucose intake assay in yeast cells. The nanoparticles observed a higher percentage of glucose intake in yeast cells nearby standard drug.

Several studies have recently shown that oxidative stress is a major contributor to the development of type 2 diabetes and its consequences. 27, 28 Dietary antioxidants play an imperative role in the management of diseases or disorders mortality and complications, including type 2 diabetes, by preventing peroxidation chain reactions. Several studies have proved that natural sources such as plants and their ingredients have a huge response to antioxidant properties without side effects. 23, 24 The bioactive compounds such as phenolic compounds, flavonoids, steroids, and vitamins are majorly present in plants, which mainly involve antioxidant activity. Total antioxidant capacity, ferrous reducing capacity, and DPPH scavenging activity are exclusive identifying markers for free radical scavenging facilities of plants extracts and their ingredients. In this study, total antioxidant, ferrous reducing capacity, and DPPH scavenging assays were utilized to determine the antioxidant mechanism of biotic nanoparticles. The total antioxidant potential of synthesized nanoparticles was observed to significantly increase in a dose dependent manner and the results were closely related to standard drug. This obtained antioxidant activity of synthesized nanoparticles with minimal dosage concentrations may be associated with the nano-sized particles of plant extract ingredients. Reducing power has been shown in several studies to be an essential quality method for determining the antioxidant activity of plants, compounds, and nanoparticles. The reducing power is closely related to the reductants, which are generally present in plant ingredients, and these exert antioxidant exploits by scavenging free radicals by donation of hydrogen. In our study, the iron reducing capacity of synthesized nanoparticles was increased depending upon the dosage concentrations. The inclusion of nano-sized biomolecules may be responsible for the reduced capability of produced nanoparticles at low dose concentrations. Scavenging of free radicals plays a delicious role in preventing disease or disorder complications, including type 2 diabetics. The DPPH activity agent generally has hydrogen donation ability and is the most accepted mechanism for in vitro antioxidant determination assay. The free radical scavenging activity of produced nanoparticles was dramatically boosted depending on the dosage concentrations. The antioxidant potential of nanoparticles has been demonstrated by their DPPH scavenging activity, which could be attributable to the presence of nano-sized biomolecules. This result indicates that the biologically synthesized nanoparticles have better glucose intake mechanisms in cells and antioxidant potential without any structural changes to the presented biomolecules.

## Conclusion

This study concluded that the nutritional flowering plant Cassia fistula is a safe and likely source for the manufacture of iron oxide nanoparticles. The purity, small size, and high solidity of the produced iron nanoparticles were validated using the indicated physical and chemical procedures. Furthermore, through the inhibitory activity of α-amylase, improved prospective intake of surplus glucose mechanism in yeast cells, and enhanced properties of total antioxidant capacity, ferrous reducing capacity, and DPPH scavenging activity, this nanoparticle acted as a potent anti-diabetic and antioxidant agent. Future studies should be conducted on the molecular mechanism of these potent anti-diabetic and antioxidant properties and also should be followed by the next level of clinical trials.
